# Childhood allergic diseases across geographical regions of Kandy and Anuradhapura districts of Sri Lanka; where do the rates stand among other regions: experience from Global asthma network Phase 1 study

**DOI:** 10.1186/s13223-022-00720-z

**Published:** 2022-08-28

**Authors:** Jagath C. Ranasinghe, Ruchira R. Karunarathne, Thilini S. Munasinghe, Gihani U. Vidanapathirana, Sanath T. Kudagammna

**Affiliations:** 1 Teaching Hospital Peradeniya, Peradeniya, Sri Lanka; 2 Principle Investigator for Anuradhapura Centre in Global Asthma Network Phase 1 Study Group, Anuradhapura, Sri Lanka; 3 Department of Paediatrics, Faculty of Medicine, University of Peradeniya, Peradeniya, Sri Lanka; 4 Principle investigator for Kandy Centre in Global Asthma Network Phase 1 Study group, Kandy, Sri Lanka

**Keywords:** Childhood asthma, Allergic rhinitis, Eczema, Global Asthma Network Phase 1, Kandy, Anuradhapura

## Abstract

**Background:**

Asthma, allergic rhinitis and eczema have been identified as the most prevalent childhood allergic diseases. However, the prevalence of these allergic diseases can vary in different regions within a country and in the world.

**Methods:**

The objective of the study was to estimate the prevalence of asthma, allergic rhinitis and eczema in schoolchildren in the Kandy and Anuradhapura districts of Sri Lanka. This was a multicentre cross sectional study carried out among children of age 6–7 years and 13–14 years attending state schools fulfilling the entry criteria of the Global Asthma Network Phase 1 study methodology.

**Results:**

A total of 3673 children of 6–7 years and 4658 children of 13–14 years were recruited. The prevalence of current asthma, allergic rhinitis and eczema were 12% (CI 10.44–13.75), 15.7% (CI 13.94–17.64) and 9.7% (CI 8.30–11.31) among 6–7 years age group and 15.3% (CI 13.66–17.09), 30.5% (CI 28.86–32.74) and 7.3% (CI 6.15–8.65) respectively among the 13–14 age group in Kandy district. The reported prevalence rates of the disease conditions were 9.9% (CI 8.72–11.22), 10.1% (CI 8.90–11.44) and 5.9% (CI 4.98–6.98) among 6–7 years age group and 14.9% (CI 13.67–16.22), 22.5% (CI 21.04–24.03) and 1.8% (CI 1.38–2.34) in the 13–14 years age group in Anuradhapura district. When comparing these prevalence rates, there is relatively a higher prevalence of childhood allergic diseases in Kandy district. This difference is statistically significant in all three allergic disease conditions (P < 0.001).

**Conclusion:**

Prevalence of allergic diseases in Anuradhapura is closer to reported data in the previous studies. There is relatively higher prevalence of childhood allergic diseases among children in Kandy district.

## Introduction

Asthma, allergic rhinitis and allergic eczema have been identified as the most prevalent childhood allergic diseases [1].The International Study on Asthma and Allergies in Childhood (ISAAC), the principal international study on paediatric allergic diseases, has revealed the prevalence patterns in different countries of the world. The highest prevalence was identified in developed countries and the lowest in some of the developing countries [2]. According to the findings of phase three, conducted at least 5 years from the first, the prevalence of allergic diseases is increasing in many countries especially in those countries shown to have lower prevalence in the phase one trial [2].

Recent research findings have shown an increasing trend in the prevalence of asthma and allergies in urban areas than in the rural areas [3]. Industrialization and the road traffic in the cities are responsible for this higher prevalence according to the literature [4]. Recent findings suggest a positive correlation between exposure to air pollution and exacerbation of asthma [5]. The incidence of allergic symptoms in children is associated with exposure to allergens in indoor environments with poor air quality [6, 7].

In addition to that, there is an association between climate changes and increasing prevalence of asthma and allergies in children. Warmer temperatures are related to increased airborne pollen and dust particles [8].

### Allergic diseases in Sri Lanka

There are several studies conducted in various districts in Sri Lanka using the ISAAC tool. Apart from few studies in the western province there is a paucity of data regarding the prevalence of childhood allergic diseases in Sri Lanka [9–11] A recent study conducted among 3–5 year- old- children in underserved settlements in Colombo Municipal Council (CMC) has revealed a prevalence of ‘ever wheezing illness’ and ‘current wheezing illness’ as 38% and 21.3% respectively [7] A study conducted in Gampaha district in Western province shows the prevalence rates for current wheezing as 16.7%,ever wheezing 19.4%, current asthma 10.7%, and physician diagnosed asthma as 14.5% [9, 12].

Air pollution has been identified as a risk factor for childhood allergic diseases in some studies. A study designed to assess the respiratory health status of 7–10 year‐old children in two settings children from an urban setting and a semi-urban setting in Sri Lanka has found the urban setting had a significantly higher prevalence of wheezing within the last 12 months as compared to children from the semi‐urban setting and also have identified poor indoor air quality as a major determinant of wheezing for the study group [13].

### Study setting

Sri Lanka is a small Island in the Indian ocean with wide variation in geographic setting, climate, urbanization and population density. It consists of central hills surrounded by low lying plains. Out of the 25 districts 6 including Kandy are predominantly in the central hills and the rest in the plains. Anuradhapuara is a region in the low lying North central province of Sri Lanka, in the dry zone, with relatively a warmer temperature. Population density of the country varies from 50 Km^2^ Mullaitivu district to 3340 Km^2^ in Colombo. Kandy is an urban region with higher population density (750 Km^2^) with a relatively low air quality index thought to be due to traffic congestion and its location [14]. Anuradhapura is less populated (population density 129 Km^2^) and has better air quality index [14]. Prevalence data on paediatric allergic diseases in regions other than Colombo and Gampaha districts is limited. The objective of the present study was to estimate the prevalence of asthma, allergic rhinitis and eczema in schoolchildren in the Kandy and Anuradhapura districts. Understanding the prevalence of childhood allergic diseases in these two districts and comparing with available data on other areas of the country will help to identify the patterns of allergic diseases in different geographical areas of the country [15]. This will help early detection of cases, optimizing the limited health resources available for the whole population. This will also feature the status of paediatric allergic diseases in the global context.

## Materials and methods

This was a multicentre cross sectional study carried out in Kandy and Anuradhapura districts as part of Global Asthma Network phase 1 study in Sri Lanka. GAN Standardized Written Core Questionnaires developed from ISAAC Questionnaires for use in phases I and III, were used in GAN phase 1 study [11]. The study sample consisted of children of age 6–7 years and 13–14 years attending state schools of Kandy and Anuradhapura districts. To fulfill the sample selection according to the GAN manual, minimum number of 10 schools with more than 1000 students from Kandy and Anuradhapura were randomly selected based on the geographic area. All children in the selected age groups in the school were recruited. Passive consent was taken from returning the completed questionnaires as stated in the study protocol. Minimum participation rate of at least 80% for adolescents and 70% for children was achieved. The questionnaires of the 6–7 year olds were completed by the parents and those of the 13–14 years were self-administered. The questionnaire was translated to Sinhala according to the Standard Operation Procedure guideline described in the manual [16]. It was validated, pre tested and shared with the GAN steering committee. The funding for the study was obtained as a research grant from the University of Peradeniya, Sri Lanka. (Ethical approval for the study was obtained from the Ethical Review Committee, Faculty of Medicine, University of Peradeniya (2018/EC/10).

### Definitions

According to the ISAAC recommendations, children with reported wheezing or whistling in the chest in the last 12 months were defined as having “current wheeze”. Children who had sneezing or a runny or blocked nose, without a “cold” or the “flu”, in the last 12 months were defined as having allergic rhinitis. Children were considered to have eczema if the responses to all of the following questions were “yes”: (1). Has your child ever had an itchy rash which was coming and going for at least 6 months?; (2). Has your child had this itchy rash at any time in the last 12 months?; (3). Has this itchy rash at any time affected any of the following places: the folds of the elbows, behind the knees, in front of the ankles, under the buttocks, or around the neck, ears or eyes?

#### Data analysis

Data entered using Epi-Info package and analyzed using the Statistical Package for the Social Sciences **(**SPSS-version 20). Statistical significance was estimated with Student’s t test and chi-square test. Significance was taken at P < *0.05.* Odd ratio and 95% confidence interval calculated to assess the risk of exposure to individual factors and presented in the tables and charts.

## Results

**Figure 1:**
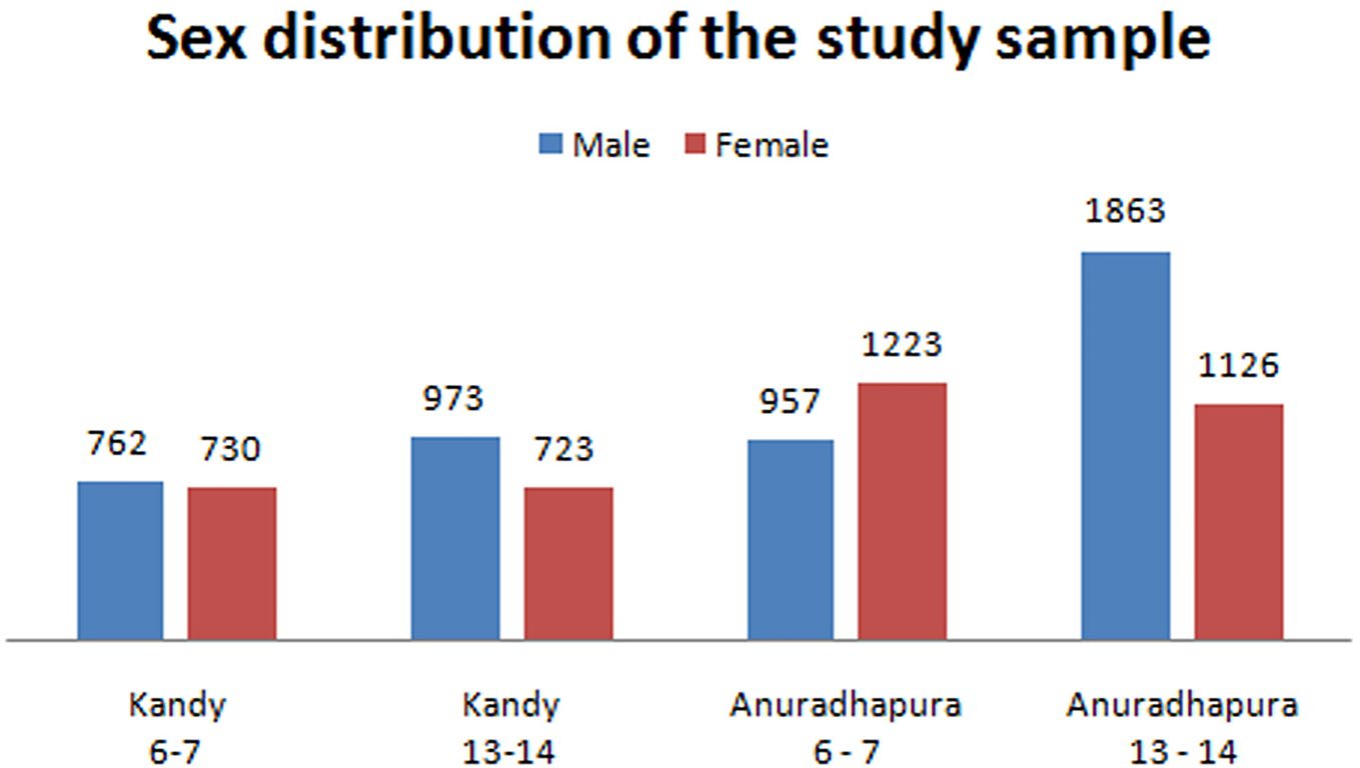
Gender distribution of the study sample

A total of 3673 children of 6–7 years (1492 children from Kandy centre and 2180 children from Anuradhapura centre) and 4658 children of 13–14 years (1696 children from Kandy centre and 2989 children from Anuradhapura centre) were recruited for the study. Gender distribution of the study cohorts have been shown in Fig.  1.

The prevalence rates for asthma, allergic rhinitis and eczema in the study groups are shown in Tables 1, 2 and 3. There were no statistically significant sex-related differences in the prevalence of these allergic diseases.

**Table 1:** Percentage of positive responses in questions related to wheezing and asthma in Kandy and Anuradhapura

	Kandy (95% CI)	Anuradhapura (95% CI)	P^***^value
6–7 Years			
Asthma ever*			
Current wheeze	12% n = 179 (10.44–13.75)	9.9% n = 217 (8.72–11.22)	0.008
Wheezing ever	18.4% n = 274 (16.57–20.52)	15.1% n = 328 (13.66–16.67)	P < 0.001
Asthma ever	8.3% n = 124 (7.0–9.81)	5.6% n = 122 (4.71–6.65)	P < 0.001
Asthma confirmed by a doctor^*^	58.1% n = 72 (49.26–66.38)	74.6% n = 91^**^ (66.16–81.52)	P < 0.001
Symptoms in past 12 months**			
Asthma attacks	97.8% n = 175 (94.20–99.33)	93.5% n = 203 (89.38–96.20)	0.034
Night Awaking	73.2% n = 131 (66.24–79.15)	53% n = 115 (46.36–59.53)	0.058
Severe wheeze	3.9% n = 7 (1.76–8.00)	11.06% n = 24 (7.49–15.98)	0.027
Exercise wheeze	95.5% n = 171 (91.29–97.86)	46.1% n = 100 (39.58–52.73)	P < 0.001
13–14 years			
Current wheeze	15.3% n = 259 (13.66–17.09)	14.9% n = 446 (13.67–16.22)	P < 0.001
Wheezing ever	21.9% n = 371 (20.00–23.93)	19.9% n = 594 (18.54–21.40)	P < 0.001
Asthma ever	12.6% n = 214 (11.11–14.27)	10.5% n = 314 (9.45–11.65)	P < 0.001
Asthma confirmed by a doctor^*^	82.2% n = 176 (76.54–86.81)	77.4% n = 243^**^ (72.4–81.68)	P < 0.001
Symptoms in past 12 months**			
Asthma attacks	86.1% n = 223 (81.33–89.82)	89.1% n = 397 (85.75–91.61)	P < 0.001
Night Awaking	44.4% n = 115 (38.48–50.49)	50.1% n = 232 (47.38–56.62)	P < 0.001
Severe wheeze	9.3% n = 24 (6.26–13.47)	11.2% n = 50 (8.59–14.49)	0.007
Exercise wheeze	38.6% n = 100 (32.89–44.67)	51.1% n = 228 (46.49–55.73)	P < 0.001

^*^Only the children who had asthma ever was considered

^**^Only the children with current wheeze was considered

^***^P value for chi square test of positive response between the two study centers

**Table 2:** Percent of positive response in questions related to allergic rhinitis in Kandy and Anuradhapura

	Kandy (95% CI)	Anuradhapura (95% CI)	P value***
6–7 Years age group			
Current Allergic Rhinitis (AR)	15.7% n = 234 (13.94–17.64)	10.1% n = 219 (8.90–11.44)	P < 0.001
Current nose symptom*	37.1% n = 87 (31.24–43.54)	45.2% n = 99 (38.75–51.82)	0.025
Current eye symptom*	32.1% n = 75 (26.40–38.29)	39.3% n = 86 (33.04–45.87)	0.015
Hay fever ever	13.2% n = 197 (11.58–15.02)	10.6% n = 231 (9.38 – 11.97)	0.572
Hay fever confirmed by a doctor^**^	74.6% n = 147 (68.09–80.20)	67.9% n = 157 (61.69 – 73.65)	P < 0.001
13–14 years age group			
Current Allergic Rhinitis (AR)	30.5% n = 517 (28.36–32.74)	22.5% n = 673 (21.04–24.03)	P < 0.001
Current nose symptom*	38.5% n = 199 (34.40–42.76)	42.1% n = 283 (38.38–45.82)	P < 0.001
Current eye symptom*	40.4% n = 209 (36.28–44.71)	50.8% n = 342 (47.05–54.58)	P < 0.001
Hay fever ever	17.5% n = 296 (15.77–19.39)	13.8% n = 414 (12.61–15.08)	0.824
Hay fever confirmed by a doctor^**^	70.3% n = 208 (64.82–75.20)	65.9% n = 273 (61.25–70.34)	P < 0.001

^*^Only the children who had current symptoms were considered

^**^Only the children who had AR ever was considered

^***^P value for chi square test of positive response between the two study centers

**Table 3:** Percent of positive response in questions related to eczema in Kandy and Anuradhapura

	Kandy (95% CI)	Anuradhapura (95% CI)	P^***^value
6–7 Years age group			
Current eczema*	9.7% n = 145 (8.30–11.31)	5.9% n = 128 (4.98–6.98)	P < 0.001
Eczema ever**	11.5% n = 172 (9.98–13.23)	9.4% n = 205 (8.29–10.76)	P < 0.001
Flexural Area	6.9% n = 104 (5.77–8.36)	3.8% n = 82 (3.05–4.67)	P < 0.001
Symptoms in past 12 months			
Rash ever**	5.9% n = 128 (3.97–5.78)	7.3% n = 123 (6.15–8.65)	P < 0.001
Rash clear^*^	54.5% n = 79 (53.07–69.69)	62.5% n = 80 (56.26–72.91)	0.711
Night awaking^*^	55.2% n = 80 (53.86–70.42)	47.6% n = 61 (40.90–58.31)	0.152
13–14 Years age group			
Current eczema*	7.3% n = 123 (6.15–8.65)	1.8% n = 55 (1.38–2.34)	P < 0.001
Eczema ever**	11.2% n = 189 (9.79–12.82)	2.4% n = 72 (1.91–3.01)	P < 0.001
Flexural Area	7.5% n = 128 (6.42–8.96)	1.1% n = 33 (0.78–1.55)	P < 0.001
Symptoms in past 12 months			
Rash ever**	5.9% n = 128 (4.98–6.98)	1.8% n = 55 (1.38–2.34)	P < 0.001
Rash clear^*^	64.2% n = 79 ( 53.07–69.69)	81.8% n = 45 (69.47–90.01)	0.135
Night awaking^*^	65.1% n = 80 (53.86–70.42)	49.1% n = 27 (36.38–61.92)	0.012

^*^Only the children with current eczema was considered

^**^Only children had eczema ever was considered

^***^P value for chi square test of positive response between the two study centers

When considering the answers to the questions related to asthma and wheezing, there is a higher prevalence of current wheeze among 6–7 (12%) and 13–14 (9.9%) years aged children in Kandy District. In addition to that, there is a significant difference of the prevalence of current wheeze (P < 0.001), wheeze ever (P < 0.001), asthma ever (P < 0.001) and symptoms in the past 12 months between the two study centers (Table 1).

There is a higher prevalence of current allergic rhinitis in Kandy among both age categories. Furthermore, the difference in the prevalence of current nose symptoms and eye symptoms is significant between the two study centers; Kandy and Anuradhapura (Table 2).

According to the answers given to the questions relevant to eczema, there is a higher prevalence of current eczema in Kandy among 6–7 and 13–14 years children which shows a significant difference between the two centers (Table 3).

### Level of control of wheezing

When considering the 6–7 years study group from Anuradhapura, 3.6% of the children had urgently been to a doctor due to breathing problems. However, only 0.2% of the children have been to an Emergency Department without being admitted to hospital because of breathing problems. In the 12–13 years study group, 9.9% of the children had been to a doctor due to a breathing problem. Among them, 1.33% has been admitted to the Emergency Department without being admitted to hospital because of breathing problems. However, these findings were not statistically significant.

When comparing above results with the study cohort from Kandy, 6.7% of the children of 6–7 years have been to a doctor due to a breathing problem while 0.9% has been admitted to an Emergency Department without being admitted to hospital because of breathing problems. In addition to that, 11.5% of the children aged 12–13 years have been to a doctor due to a breathing problem. Among them, 1.4% has been admitted to an Emergency Department without being admitted to hospital because of breathing problems.

## Discussion

In this multicenter cross sectional study, We found that the prevalence of current asthma, allergic rhinitis and eczema were 12%, 15.7% and 9.7% among 6–7 years age group in Kandy district while it was 15.3%, 30.5% and 7.3% respectively among the 13–14 age group. The reported prevalence rates of current asthma, allergic rhinitis and eczema were 9.9%, 10.1% and 5.9% among 6–7 years age group in Anuradhapura district. In addition to that, the prevalence rates of current asthma, allergic rhinitis and eczema were 14.9%, 22.5% and 1.8% in the 13–14 years age group from Anuradhapura district. When comparing these prevalence rates, there is relatively a higher prevalence of childhood allergic diseases in Kandy district. This difference is statistically significant for all three allergic disease conditions (P < 0.001).

There is a difference in asthma confirmed by a doctor in Kandy and Anuradhapura districts while it is not as such for others. This seems allergic diseases being under diagnosed in some areas which warrant further evaluation.

There are only a few local studies on the prevalence of allergic diseases in children of similar age groups. A similar study conducted in the western province has revealed a prevalence of asthma as 17%, Allergic rhinitis 21.4%, and eczema as 5% in a representative sample of children studying in grade 5 (age 10 years)[16]. When comparing these results with the present study, Anuradhapura district results are in parallel with the western province results while there is a deviation in Kandy district results. Another study conducted in Chilaw area (Gampaha district from western province) of Sri Lanka has reported a 17% of asthma prevalence rates and 22% of allergic rhinitis [17]. These results indicate there is a diversity of the prevalence rates among different geographic regions.

In our study, the overall prevalence of current allergic rhinitis in the 6–7 year and 13–14 year age groups are 12.9%, 26.5% respectively. It is higher than the mean of global prevalence (9.1%, 16%), and the Asia–Pacific prevalence of AR (5.8% and 14.5%) [14]. In addition to that, the overall prevalence of current eczema symptoms in the 6–7 years and 13–14 years age groups are 7.8%, 4.6% respectively. There are certain deviations when comparing these findings with the mean global prevalence (7.9%, 7.3%) and the Asia–pacific prevalence of eczema (4.7% and 5.3%) [3].

When comparing our results with other countries in the world, the developed countries have reported high prevalence of allergic diseases. Asher et al. have revealed a range of 20–37.6% prevalence of childhood allergic diseases in England, Australia and certain regions of US [1]. However, the rates are lower in the Asian region; India 6.4%, Pakistan 11.7%, Indonesia 5.2% [1].

The reported prevalence of wheeze in the present study is relatively high when comparing with other countries in the region; India 6.4%, Pakistan 11.7%, Indonesia 5.2% [1]. The reported prevalence of allergic rhinitis is slightly higher than that reported from the countries in the region: India 10%, Indonesia 4.8% and more or less similar to Pakistan 16.8%. With respect to eczema, Sri Lanka appears to have an approximately similar prevalence to India (3.7%) but lower than that of Pakistan (13.2%) [1].

The difference of the prevalence rates of allergic diseases in the two districts might be attributed to several factors. Kandy is an urban city where there is a poor air quality index due to the geographical location and traffic congestion [14]. This might be responsible for the higher prevalence of allergic diseases among the study groups than to Anuradhapura. A recent study conducted in China has also revealed that climatic variation and air pollution were associated with childhood allergic diseases [18]. In contrast, Anuradhapura has a relatively cleaner air quality [14]. Also Kandy is situated in the wet zone of the country while Anuradhapura situated in the dry zone of the country (Fig. 2). This climate changes may also need to be considered. In addition to that, urbanization, temperature, humidity, and regional differences could be linked to variations in prevalence [19]. On the other hand Kandy being second city in Sri Lanka and being an urban city, it has more accessible medical institutions around the clock compared to Anuradhapura being a rural district. Also road and accessibility is higher in Kandy vs Anuradhapura. However, further research is essential to support these speculations.

### Limitations of the study

Limited response rate for certain questions in the questionnaire was identified as a limitation of the present study. This can be attributed mainly due to conducting the study during unusually harsh monsoon season and during post- year end-examinations where student attendance is generally low. Self-reporting for 13/14 year olds is a potential confounder, as is the inability to distinguish AR from NAR in this study. NAR might be more prevalent in polluted areas as well, further compounding this issue.

## Conclusion

In conclusion, the results of the present study revealed a relatively higher prevalence of asthma, AR and eczema in Kandy compared to Anuradhapura. Further focused studies should be conducted to identify the reason for high prevalence compared to the other countries in the region with etiological agents of allergic diseases in children.

## Data Availability

All necessary data is available in the manuscript. Study materials available from GAN official website.

## Abbreviations

AR: Allergic rhinitis
CMC: Colombo Municipal Council
GAN: Global asthma network
ISAAC: The International Study on Asthma and Allergies in Childhood
NAR: Non allergic rhinitis
SPSS: Statistical package for the social sciences
